# Comparison of venous and pooled capillary hemoglobin levels for the detection of anemia among adolescent girls

**DOI:** 10.3389/fnut.2024.1360306

**Published:** 2024-06-06

**Authors:** Mica Jenkins, Esi Foriwa Amoaful, Mutala Abdulai, Veronica Quartey, Ruth Situma, Porbilla Ofosu-Apea, Jevaise Aballo, Maku E. Demuyakor, Lucas Gosdin, Carine Mapango, Maria Elena D. Jefferds, O. Yaw Addo

**Affiliations:** ^1^Division of Nutrition, Physical Activity, and Obesity, National Center for Chronic Disease Prevention and Health Promotion, Centers for Disease Control and Prevention (CDC), Atlanta, GA, United States; ^2^Nutrition and Health Sciences Doctoral Program, Laney Graduate School, Emory University, Atlanta, GA, United States; ^3^Nutrition Department, Family Health Division, Ghana Health Service, Ministry of Health, Accra, Ghana; ^4^UNICEF, Accra, Ghana; ^5^Hubert Department of Global Health, Rollins School of Public Health, Emory University, Atlanta, GA, United States

**Keywords:** hemoglobin, anemia, blood source, capillary, venous, adolescents

## Abstract

**Introduction:**

Blood source is a known preanalytical factor affecting hemoglobin (Hb) concentrations, and there is evidence that capillary and venous blood may yield disparate Hb levels and anemia prevalence. However, data from adolescents are scarce.

**Objective:**

To compare Hb and anemia prevalence measured by venous and individual pooled capillary blood among a sample of girls aged 10–19 years from 232 schools in four regions of Ghana in 2022.

**Methods:**

Among girls who had venous blood draws, a random subsample was selected for capillary blood. Hb was measured using HemoCue® Hb-301. We used Lin’s concordance correlation coefficient (CCC) to quantify the strength of the bivariate relationship between venous and capillary Hb and a paired t-test for difference in means. We used McNemar’s test for discordance in anemia cases by blood source and weighted Kappa to quantify agreement by anemia severity. A multivariate generalized estimating equation was used to quantify adjusted population anemia prevalence and assess the association between blood source and predicted anemia risk.

**Results:**

We found strong concordance between Hb measures (CCC = 0.86). The difference between mean venous Hb (12.8 g/dL, ± 1.1) and capillary Hb (12.9 g/dL, ± 1.2) was not significant (*p* = 0.26). Crude anemia prevalence by venous and capillary blood was 20.6% and 19.5%, respectively. Adjusted population anemia prevalence was 23.5% for venous blood and 22.5% for capillary (*p* = 0.45). Blood source was not associated with predicted anemia risk (risk ratio: 0.99, 95% CI: 0.96, 1.02). Discordance in anemia cases by blood source was not significant (McNemar *p* = 0.46). Weighted Kappa demonstrated moderate agreement by severity (*ĸ* = 0.67). Among those with anemia by either blood source (*n* = 111), 59% were identified by both sources.

**Conclusion:**

In Ghanaian adolescent girls, there was no difference in mean Hb, anemia prevalence, or predicted anemia risk by blood source. However, only 59% of girls with anemia by either blood source were identified as having anemia by both sources. These findings suggest that pooled capillary blood may be useful for estimating Hb and anemia at the population level, but that caution is needed when interpreting individual-level data.

## Introduction

Anemia is a common blood condition and a global health problem that affects approximately 40% of children aged 6–59 months, 30% of nonpregnant women aged 15–49 years, and 36% of pregnant women aged 15–49 years (1). Iron deficiency is a key cause of anemia (2, 3), and evidence has shown that 25% of anemia cases in preschool children and 37% in women of reproductive age are attributable to iron deficiency (4). The World Health Organization (WHO) has established population prevalence ranges to indicate the magnitude of iron deficiency and anemia as public health problems, with prevalence of 5–19.9% considered mild, 20–39.9% considered moderate, and 40% and above considered high (3, 5). These ranges exist alongside recommendations for iron supplementation in school-age children and menstruating women in settings where the prevalence of anemia is 20% or higher (6, 7). The consequences of iron deficiency and anemia include adverse pregnancy outcomes (i.e., low birth weight, prematurity, and increased risk of maternal illness and mortality), compromised growth, suppressed immune function, impaired cognitive development, and loss of individual and national productivity (8–11). The prevention and detection of anemia in adolescent girls is of particular importance due to the increased need for micronutrients during this developmental life stage, with estimated average requirements for iron increasing by nearly 40% between the ages of 13 and 14 years (11, 12).

Anemia is diagnosed by comparing hemoglobin (Hb) levels, which can be measured in venous or capillary blood, to globally accepted thresholds (3, 13, 14). Depending on the context, either or both blood sources may be used to estimate population-level anemia prevalence and/or to screen for anemia at the individual level. Venous blood assessed by automated hematology analyzer has been established as the gold standard for anemia diagnosis (14, 15). However, due to the considerable cost and logistic demand of collecting venous blood (i.e., strict cold chain requirements for transportation and storage), governments and their partners must weigh the advantages of using the reference method against the significant inputs needed to successfully conduct national-scale health surveys. Collecting capillary blood via finger prick requires less training, uses only a small volume of blood, and does not require cold storage if analyzed immediately using a point-of-care hemoglobinometer (16). In real-world settings of Hb measurement, the single drop method is more commonly used than pooled capillary blood as it is relatively quicker to implement and requires fewer materials (e.g., microtubes, pipettes, and parafilm).

Blood source is a known preanalytical factor affecting Hb levels and anemia prevalence, alongside postural effect (i.e., sitting vs. standing), and environmental factors such as temperature and humidity (17). In a 2019 review, Whitehead et al. identified 12 studies which found higher mean Hb in capillary blood compared to venous blood, and three studies which found lower Hb levels in capillary blood compared to venous (17). In this same review, the authors identified four studies which compared single drop capillary blood to pooled capillary blood, finding distinct differences. Such inconsistencies in Hb levels by blood source translate into discrepancies in anemia diagnosis, which in turn influence clinical decisions and the interpretation of the anemia burden, intervention response, and program decision-making (14). A recent review of survey data from multiple countries found that anemia prevalence estimates were consistently lower in surveys using venous blood compared to capillary (18).

A limited number of studies have compared pooled capillary blood to venous blood for the measurement of Hb and detection of anemia. A recent study in Mexican children and adults found that venous and pooled capillary blood performed comparably in the determination of Hb levels (19), and a study among Indian women found that Hb estimates and anemia prevalence were not significantly different using pooled capillary blood compared to venous blood (20). Similarly, two earlier studies found that pooled capillary blood, compared to single drop, improved the precision of Hb measurement compared to estimates obtained using venous blood (21, 22). Although multiple studies have compared Hb level and/or anemia prevalence by blood source among young children and adults (14, 18–25), our literature review yielded no publications that have made such comparisons in adolescents aged 10–19 years.

Our objective is to compare Hb levels and anemia prevalence among adolescent girls in Ghana as detected by venous and individual pooled capillary blood and to ascertain if blood source is associated with adjusted population anemia risk. Such information could guide decision-making around preferred methods for estimating Hb for screening and clinical purposes, and for monitoring the anemia situation in-country to inform timely public health interventions.

## Methods

### Data collection and point-of-care procedures

**Figure 1:**
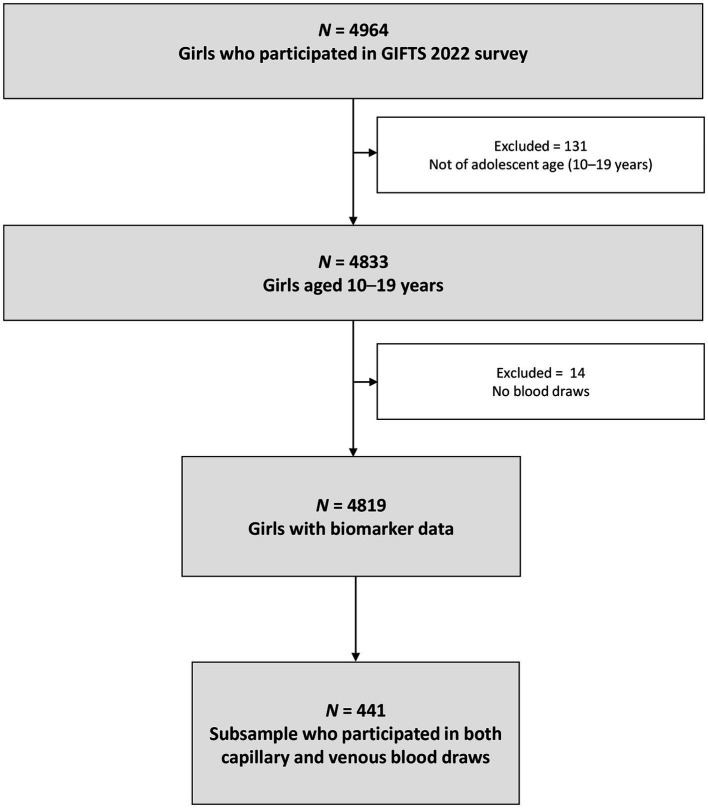
Participant flow chart of school-going adolescent girls from a nationally representative survey in Ghana, 2022. GIFTS, Girls’ Iron-Folate Tablet Supplementation Program.

We collected data in 2022 from 4,833 adolescent girls aged 10–19 years in Ghana participating in the government-led Girls’ Iron-Folate Tablet Supplementation (GIFTS) program (26) (Figure 1). We used a multistage complex survey design in which schools (clusters) were selected by probability proportional to size sampling, from four ethnically and geographically diverse regions spanning three ecologic zones (strata), to achieve a nationally representative sample of school-going adolescents. Venous blood draws were obtained by trained phlebotomists from 4,819 girls. A random subsample of 441 girls was selected for additional individual pooled capillary blood draws. The HemoCue® Hb-301 System was used by phlebotomists to determine Hb status using venous blood at the point of care as well as pooled capillary blood among those in the subsample. Anemia status and severity were defined using the WHO Hb concentrations for the diagnosis of anemia (3). All girls who tested positive for malaria and/or had anemia were given a referral to seek medical care at a health facility per Ghana Ministry of Health protocol.

### Statistical analyses

Individual-level data were analyzed and pooled to estimate population mean Hb and anemia prevalence from both venous and pooled capillary blood. We used a Bland–Altman plot to evaluate agreement between continuous venous and capillary Hb, Lin’s concordance correlation coefficient (CCC) to quantify the strength of the bivariate relationship, and a paired t-test for difference in population mean Hb. In cross-tabulation analysis, we calculated agreement statistics (i.e., sensitivity, specificity, and accuracy) to determine the appropriateness of capillary blood for diagnosing anemia compared to venous blood as the reference method. McNemar’s test was conducted to assess discordance in anemia cases identified by each blood source, and weighted Kappa was used to quantify agreement between the two at varying levels of anemia severity. We used a multivariate generalized estimating equation (GEE) with identity link function to quantify adjusted population anemia prevalence and assess if blood source is associated with the predicted risk of anemia (27). This model accounted for two blood measurements from each adolescent girl (1 venous, 1 pooled capillary) and multiple students from each school as random effects, as well as potential confounders (i.e., child age, school level, and region) as fixed effects. Covariance decomposition was used to partition intra-cluster correlation coefficients (ICC) and determine the variance in anemia prevalence due to intra-individual differences vs. phlebotomist technique (intra-school differences). All analyses were performed in SAS version 9.4 (SAS Institute Inc., Cary, NC) and R Studio version 4.3.1. Statistical significance was set at a two-sided alpha level of 0.05.

## Results

### Basic characteristics

Among the subsample of 441 girls, the mean age was 16.4 (± 1.7) years and most girls (92.5%) had reached menarche (Table 1). The majority of girls attended school in urban areas (62.6%). Awareness of anemia was moderate (64.4%), and 10 girls (3.5%) reported that they had ever had anemia. A comparison of participant characteristics between the random subsample with capillary blood draw (*n* = 441) and those without (*n* = 4,378) suggested no selection bias (Table 1). The only significant difference observed between the two samples was for venous Hb, and the magnitude of difference was small (0.2 g/dL).

**Table 1:** Basic characteristics of school-going adolescent girls from a nationally representative survey in Ghana, 2022.

	Subsample with capillary blood draw (*n* = 441)		Sample without capillary blood draw (*n* = 4,378)		Overall sample (*N* = 4,819)		*p*-value^a^
	*n*	Mean ± SD/% (95% CI)	*n*	Mean ± SD/% (95% CI)	*n*	Mean ± SD/% (95% CI)	
Age, years	441	16.4 ± 1.7	4,378	16.4 ± 1.8	4,819	16.4 ± 1.8	0.91
Post-menarche	408	92.5 (90.1, 95.0)	4,043	92.3 (91.6, 93.1)	4,451	92.4 (91.6, 93.1)	0.90
School level							
Junior high school	222	50.3 (45.7, 55.0)	2,152	49.2 (47.7, 50.6)	2,374	49.2 (47.9, 50.7)	0.64
Senior high school	219	49.7 (45.0, 54.3)	2,226	50.8 (49.4, 52.3)	2,445	50.7 (49.3, 52.1)	0.64
School location							
Rural	128	29.0 (24.8, 33.3)	1,230	28.1 (26.8, 29.4)	1,358	28.2 (26.9, 29.5)	0.68
Peri-urban	37	8.4 (5.8, 11.0)	366	8.4 (7.5, 9.2)	403	8.4 (7.6, 9.1)	0.98
Urban	276	62.6 (58.1, 67.1)	2,782	63.5 (62.1, 65.0)	3,058	63.5 (62.1, 64.8)	0.69
Heard of anemia	284	64.4 (59.9, 68.9)	2,643	60.4 (58.9, 61.8)	2,927	60.7 (59.4, 62.1)	0.10
Ever had anemia (self-report)^b^	10	3.5 (1.4, 5.7)	142	5.4 (4.5, 6.2)	152	5.2 (4.4, 6.0)	0.26
Venous Hb (g/dL)	441	12.8 ± 1.1	4,378	12.6 ± 1.2	4,819	12.7 ± 1.2	0.03
Venous anemia	91	20.6 (16.8, 24.4)	984	22.5 (21.2, 23.7)	1,075	22.3 (21.1, 23.5)	0.38

Estimates are unweighted to ensure comparability between the overall sample and subsample. SD, standard deviation; CI, confidence interval. ^a^*p*-values represent a comparison of the subsample with capillary blood draws (*n* = 441) to those without capillary blood draws (*n* = 4,378). ^b^Among those who had heard of anemia.

### Hemoglobin

**Figure 2:**
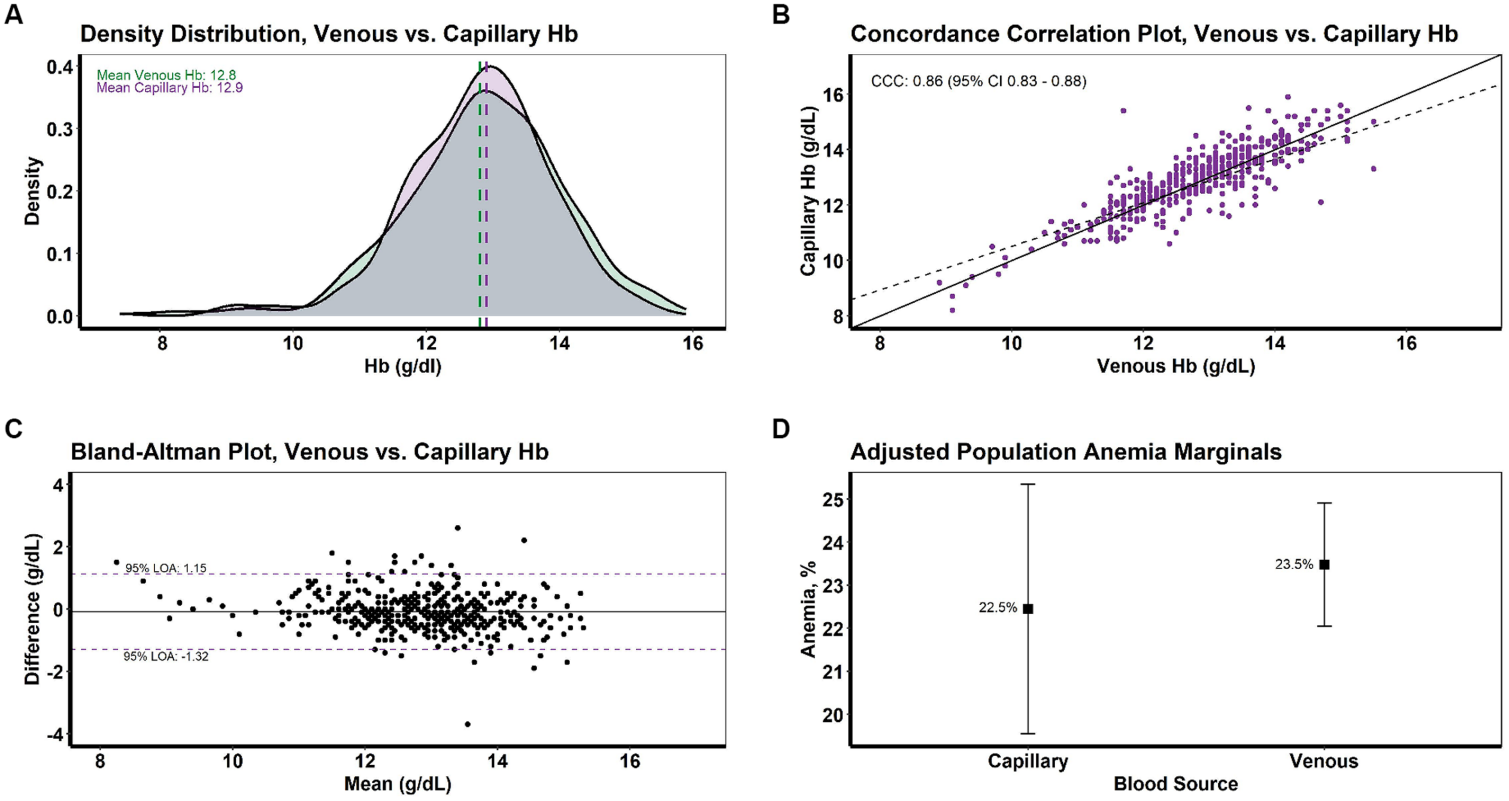
Intra-individual concordance of venous and pooled capillary hemoglobin levels and anemia among school-going adolescent girls from a nationally representative survey in Ghana, 2022 (*N* = 441). Comparisons shown include **(A)** density distribution for Hb, **(B)** concordance correlation plot for Hb, **(C)** Bland-Altman plot for Hb, and **(D)** adjusted population anemia marginals. Hb, hemoglobin; CCC, concordance correlation coefficient; LOA, limit of agreement.

A density distribution plot indicated a complete overlap of Hb distributions with comparable means; venous Hb was 12.8 g/dL (± 1.1) while capillary Hb was 12.9 g/dL (± 1.2) (Figure 2; Table 2). No statistically significant difference in mean venous and pooled capillary Hb was observed (paired t-test, *p* = 0.26). From Bland–Altman analysis, the population mean difference in Hb was very low at −0.09 g/dL with 95% limits of agreement of −1.32 and + 1.15 g/dL (Figure 2). Further, CCC analysis demonstrated strong concordance between the two Hb measures (CCC = 0.86, precision = 0.86, accuracy = 0.99).

**Table 2:** Comparison of hemoglobin levels and anemia prevalence by blood source among school-going adolescent girls from a nationally representative survey in Ghana, 2022 (*N* = 441).

	Venous		Capillary		Measure of agreement/Difference
	*n*	Mean ± SD/Proportion (95% CI)	*n*	Mean ± SD/Proportion (95% CI)	
Mean Hb (g/dL) ± SD	441	12.8 ± 1.1	441	12.9 ± 1.2	CCC = 0.86, precision = 0.86, accuracy = 0.99^a^ *T*-test, *p* = 0.26
Anemia	91	20.6 (16.8, 24.2)	86	19.5 (15.8, 23.2)	McNemar, *p* = 0.46 Weighted Kappa, *ĸ* = 0.67
Mild	67	15.2 (11.8, 18.6)	61	13.8 (10.6, 17.1)	
Moderate	23	5.2 (3.1, 7.3)	24	5.4 (3.3, 7.6)	
Severe	1	0.2 (0.0, 0.7)	1	0.2 (0.0, 0.7)	
Anemia^b^	91	20.6 (16.8, 24.2)	86	19.5 (15.8, 23.2)	Sensitivity: 0.73 Specificity: 0.94 Accuracy: 0.90

SD, standard deviation; CI, confidence interval; CCC, concordance correlation coefficient. ^a^The CCC contains both precision and accuracy measures, where precision represents the Pearson correlation coefficient and accuracy represents a bias correction factor which measures how far the best-fit line deviates from the line of origin. ^b^Agreement statistics were calculated in cross-tabulation analysis using venous blood as the reference method.

### Anemia

**Figure 3:**
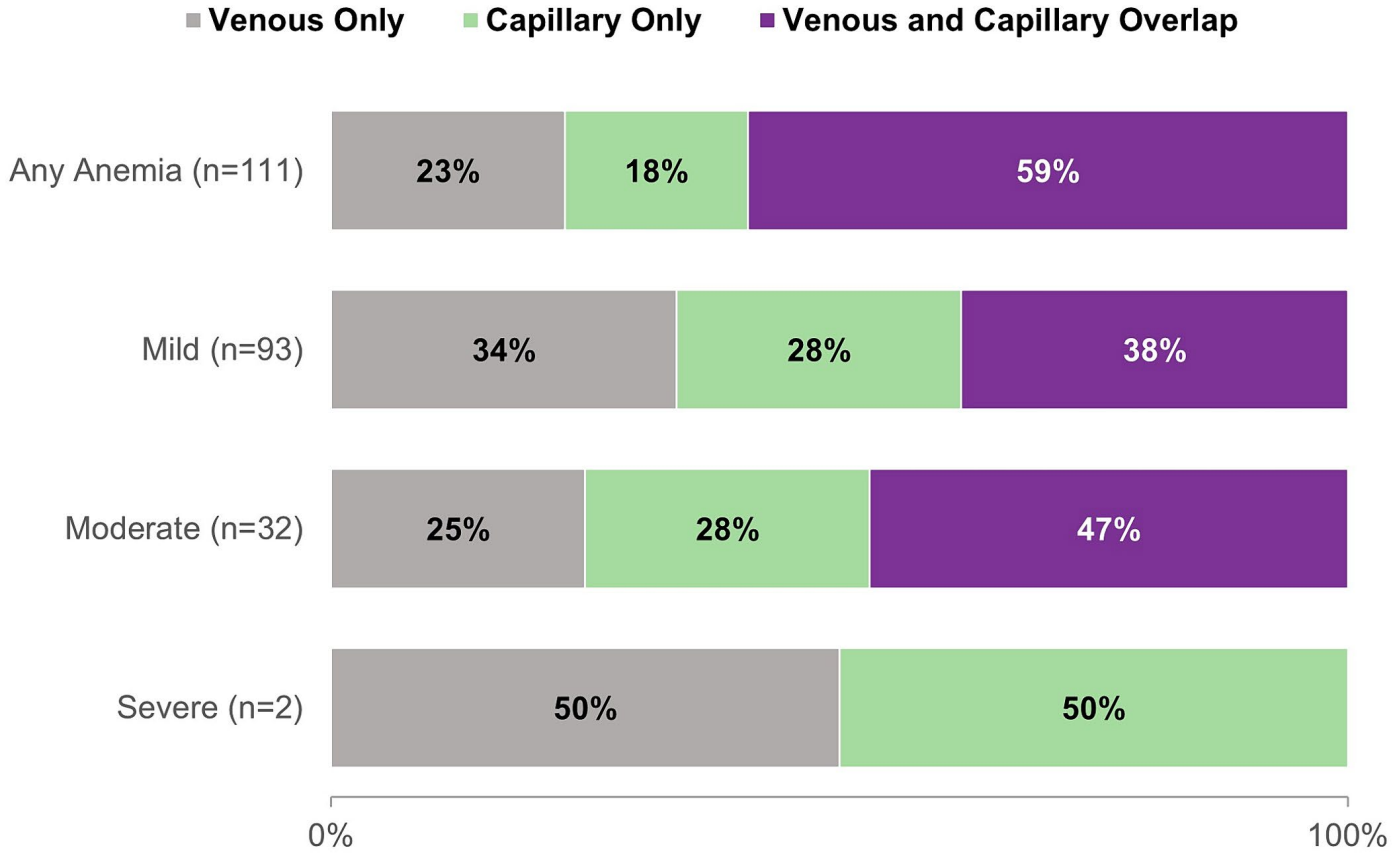
Proportion of anemia cases diagnosed by venous and pooled capillary Hb for any anemia and by severity among school-going adolescent girls from a nationally representative survey in Ghana, 2022 (*N* = 111).

The crude prevalence of anemia by venous and pooled capillary blood was 20.6% (91 of 441 girls) and 19.5% (86 of 441 girls), respectively, and the majority of cases were mild (Table 2). The sensitivity and specificity of capillary blood for diagnosing anemia compared to venous blood were 0.73 and 0.94, respectively, while accuracy was 0.90. The discordance in anemia cases identified by the two blood sources was not significant (McNemar’s test, *p* = 0.46). A total of 111 anemia cases were identified by either blood source, and among these cases, 66 (59%) were identified by both sources (Figure 3). By anemia severity—mild (*n* = 93), moderate (*n* = 32), and severe (*n* = 2)—38, 47, and 0% of cases were identified by both blood sources, respectively (Figure 3). Weighted Kappa demonstrated moderate agreement by severity (*ĸ* = 0.67). Because only two cases were identified as severe anemia (one by each blood source), we calculated a second weighted Kappa after combing moderate and severe cases and found that the outcome was not meaningfully different (*ĸ* = 0.68) (28).

Our GEE model to assess the association between blood source and risk of anemia, controlling for child age, school level, and geographic region as fixed effects, yielded an adjusted anemia prevalence of 23.5% for venous blood and 22.5% for capillary, a difference which was not significant (*p* = 0.45) (Figure 2). Blood source was not associated with the predicted risk of anemia (risk ratio: 0.99, 95% confidence interval: 0.96, 1.02). Covariance analysis indicated that intra-individual differences accounted for the majority of variance in anemia prevalence (ICC: 0.67), while school effect or phlebotomist technique accounted for minimal variance (ICC: 0.02). The remaining 31% of variance resulted from fixed effect variables, i.e., child age, school level, and geographic region of survey.

## Discussion

In our sample of Ghanaian adolescent girls, there was no difference in mean Hb or anemia prevalence as estimated by venous and pooled capillary blood, suggesting that blood source is not a factor in determining anemia status in this population. These findings are consistent with recent studies in Mexico and India which also found that pooled capillary blood and venous blood performed similarly for determining Hb and detecting anemia (19, 20). In linear analysis, we found that blood source was not associated with anemia risk, with adjusted population anemia prevalence differing by a single percentage point. However, despite substantial agreement between the two blood sources (e.g., sensitivity of 0.73 and accuracy of 0.90 with venous blood as the reference method), we found that they do not detect the same girls as having anemia; only 59% (66 of 111) of anemia cases identified by either blood source were identified by both sources (Figure 3). This finding may have practical implications for researchers and public health workers seeking the most appropriate method for screening and diagnosing iron deficiency and anemia. While pooled capillary blood may be appropriate for population-level analyses, the precision offered by venous blood measures may be preferrable for measuring Hb and diagnosing anemia at the individual level. Decision-makers must weigh the operational feasibility of implementing gold standard procedures compared to those that are simpler and less expensive to execute.

Our analysis of anemia prevalence by blood source and severity showed only moderate agreement (*ĸ* = 0.67), with less than half of cases identified by either blood source being identified by both sources for all levels of severity. This finding is likely driven by the difference in mild vs. moderate anemia diagnosis by blood source, as the two categories are distinguished by a difference in Hb of only 0.1 g/dL (3).

Historically, a single drop of capillary blood has been used to assess anemia in the Demographic and Health Survey (DHS). The 2014 and 2022 DHS in Ghana found an anemia prevalence of 48% and 44% among girls aged 15–19, respectively (29, 30). Yet, the Ghana National Micronutrient Survey conducted in 2017 using venous blood from nonpregnant women 15–49 years revealed an anemia prevalence of 26% among adolescent girls aged 15–19 (31), substantially lower than the DHS findings in the same age group. Such discrepancies create doubt regarding the use of capillary blood for population surveys. The findings from our study have suggested that, with quality training on technique, pooled capillary blood may be a viable alternative to single drop in Ghana and could be relevant to other resource-constrained settings where it is not feasible to incorporate venous blood draws into clinical settings, annual surveys, and surveillance systems. We found that only 2% of variance in intra-individual anemia prevalence was attributable to phlebotomist technique, an encouraging result suggesting strong capacity within existing public health service platforms in Ghana to collect high-quality biological data.

## Strengths and limitations

The strengths of our study include a large nationally representative sample of school-going Ghanaian adolescent girls from whom multiple biological samples were collected, enabling adjustment for intra-individual variation while isolating the role of blood source on population Hb levels and anemia prevalence. Further, our comparison of the random subsample of girls with capillary blood draw to those without suggested no selection bias, and thus our findings may be generalizable to the broader population of school-going adolescent girls in Ghana. However, as our data are from a specific population in a single country, findings should be interpreted cautiously and might not be applicable to other population groups, even within Ghana.

## Conclusion

Among Ghanaian adolescent girls, there was no difference in mean Hb, anemia prevalence, or predicted anemia risk by blood sample collection method. However, there was some discrepancy in anemia cases identified by venous and pooled capillary blood at the individual level, for any anemia and by severity. These findings suggest that, while pooled capillary blood may be useful for estimating Hb and anemia at the population level among Ghanaian adolescent girls, the same might not be true for individual-level analyses. Future efforts in this context should involve careful consideration of goals and objectives to inform the choice of blood sample collection method.

## Data availability statement

The raw data supporting the conclusions of this article will be made available by the authors, without undue reservation.

## Ethics statement

The studies involving humans were approved by the Ethical Review Committee of the Ghana Health Service. The studies were conducted in accordance with the local legislation and institutional requirements. Written informed consent for participation in this study was provided by the participants’ legal guardians/next of kin.

## Author contributions

MiJ: Resources, Supervision, Writing – review & editing, Writing – original draft, Visualization, Software, Project administration, Methodology, Investigation, Funding acquisition, Formal analysis, Data curation, Conceptualization. EA: Resources, Writing – review & editing, Supervision, Project administration, Investigation, Conceptualization. MA: Supervision, Resources, Writing – review & editing, Project administration, Investigation, Conceptualization. VQ: Writing – review & editing, Supervision, Project administration, Investigation, Resources, Conceptualization. RS: Resources, Writing – review & editing, Project administration. PO-A: Resources, Writing – review & editing, Project administration, Investigation. JA: Resources, Writing – review & editing, Project administration, Investigation. MD: Writing – review & editing, Project administration, Investigation. LG: Writing – review & editing, Project administration, Conceptualization. CM: Writing – review & editing, Investigation. MaJ: Resources, Project administration, Writing – review & editing, Supervision, Conceptualization. OA: Writing – review & editing, Writing – original draft, Visualization, Supervision, Software, Resources, Project administration, Methodology, Investigation, Funding acquisition, Formal analysis, Data curation, Conceptualization.
